# Parental awareness of screen use and myopia risk in children: a study among Turkish parents

**DOI:** 10.3389/fpubh.2026.1831752

**Published:** 2026-05-25

**Authors:** Merve Bozkurt Gençer, Kübra Küçükiba, Ayşe Cengiz Ünal, Berna Dogan, Muhammet Kazim Erol

**Affiliations:** 1 Ophthalmology Department, Antalya Training and Research Hospital, Antalya, Türkiye; 2 Ophthalmology Department, Ankara Training and Research Hospital, Ankara, Türkiye

**Keywords:** knowledge level, myopia, myopia control, questionnaire, screen time

## Abstract

**Purpose:**

In recent years, the prevalence of myopia in children has increased significantly, with increasing screen exposure considered a potential contributing factor. This study aimed to evaluate parents’ understanding of the relationship between myopia and screen time, as well as general awareness of myopia in society. In addition, parents’ knowledge of protective factors and treatment options was assessed to provide evidence for future preventive strategies and healthcare planning.

**Methods:**

A structured questionnaire was administered to 2,031 parents to collect data on demographic characteristics, children’s daily screen time, types of digital devices and content used, time spent outdoors, parental concerns regarding screen exposure, and knowledge of myopia prevention and control strategies. Associations between parental awareness and children’s behavioral patterns were analyzed.

**Results:**

The prevalence of myopia among children was 11.8%. A positive family history was the strongest factor associated with myopia, being associated with a 2.3-fold higher likelihood (*p* < 0.001). Each additional hour of daily screen exposure was associated with a 15% increase in myopia risk, whereas each additional hour spent outdoors was associated with a 28% reduction in likelihood (*p* = 0.002). Although 87% of parents reported basic awareness of myopia, knowledge of protective measures and treatment options was limited. In addition, 55.5% of children exceeded the recommended daily screen time. Higher parental education level was associated with greater adherence to regular eye examinations and more frequent adoption of protective behaviors.

**Conclusion:**

Parental knowledge alone, in the absence of corresponding behavioral changes and supportive structural interventions, was found to be insufficiently associated with reduced risk of childhood myopia. Higher parental education levels were associated with better adherence to regular eye examinations and more frequent adoption of protective behaviors. These findings suggest that strengthening parental education, expanding school-based vision screening programs, and promoting outdoor activity at the community level may be associated with improved awareness and healthier behavioral patterns related to myopia prevention.

## 1 Introduction

Children and adolescents worldwide, particularly in East Asia, are at an increased risk of developing myopia (1). In China, the prevalence of myopia among children is as high as 53.6%, and similar increasing trends are being observed in other countries (2). It is estimated that by 2050, approximately 50% of the global population may be affected by myopia (3). In most cases, myopia results from abnormal axial elongation of the eye, causing light to focus in front of the retina, which leads to reduced visual acuity. According to World Health Organization (WHO) guidelines, myopia is classified based on spherical equivalent refractive error: a value of ≤ −0.50 D in both eyes is considered myopia, whereas ≤ −5.00 D is classified as high myopia (4). Myopia increases the risk of several ocular diseases, including retinal detachment, myopic maculopathy, cataract, and glaucoma (5–7). The prevalence of sight-threatening disorders is particularly high in individuals with high myopia (8). Notably, myopic maculopathy has been reported as a leading cause of vision impairment and blindness in young and middle-aged adults in northern China (9).

Although myopia progression cannot be reversed after onset, specific interventions can delay progression to high myopia and consequently reduce the risk of myopia-related complications (8). Myopia control strategies can be broadly categorized as behavioral, optical, or pharmacological (10). Behavioral interventions, such as increasing time spent outdoors and reducing near work, are effective in preventing myopia onset but have limited impact on slowing the progression of existing myopia (11–15). The efficacy of various optical (16, 17) and pharmacological interventions (16, 18, 19), including corrective spectacles, orthokeratology, multifocal contact lenses, and high- or low-concentration atropine, has been demonstrated in multiple studies. Screen time has emerged as an important risk factor for myopia progression. Previous studies indicate that daily screen exposure of less than 1 h is generally safe, whereas 1–4 h of screen time significantly increases the risk of myopia progression (20).

Parental awareness and adherence to recommended interventions are critical, as parents play a central role in implementing behavioral, optical, and pharmacological strategies for their children. Therefore, understanding parents’ knowledge, attitudes, and practices regarding myopia and its management is essential. This study aimed to assess the relationship between myopia and screen time, as well as parental awareness of myopia risk factors and control strategies. The findings are intended to provide guidance for ocular health specialists and support effective parental involvement in follow-up and treatment.

## 2 Materials and methods

Approval for this cross-sectional, descriptive study was obtained from the Human Ethics Committee of Antalya Training and Research Hospital (decision no: 14/16, dated 28/04/2025). All study procedures were conducted in accordance with the Helsinki Declaration. Clinical trial registration was not applicable. Between May 2025 and August 2025, an online questionnaire was administered to 2,031 parents of children aged 3–17 years who were involved in the child’s daily care. The online survey link was distributed randomly to parents, and each participant was allowed to complete the questionnaire for only one child. Participation was voluntary. The questionnaire was distributed via a secure online link, and responses were automatically collected and screened by the research team. No financial support was received for this study, including research, authorship, or publication.

### 2.1 Questionnaire development

The questionnaire was developed following a comprehensive review of the literature on childhood myopia, screen exposure, and parental awareness. The initial draft included items assessing demographic characteristics, children’s screen exposure habits, duration of outdoor activities, history of eye examinations, and parental knowledge regarding myopia, its risk factors, and preventive strategies. To ensure content validity, the preliminary questionnaire was evaluated by a panel of five experts in ophthalmology. Each item was reviewed for relevance, clarity, and comprehensiveness, and minor modifications were made based on expert feedback to enhance clarity and appropriateness.

### 2.2 Pilot testing

A pilot study was conducted with 50 parents to evaluate the comprehensibility and feasibility of the questionnaire. Participants provided feedback on the clarity and interpretation of each question. Necessary revisions were made accordingly. Data from the pilot phase were not included in the final analysis.

### 2.3 Questionnaire structure

The final questionnaire consisted of three sections:

1. *Demographic information*: parents’ and children’s demographic characteristics.
2. *Screen exposure and outdoor activities*: daily screen time, types of digital devices used, and duration of outdoor activities.
3. *Parental awareness*: knowledge of myopia risk factors, preventive strategies, and potential complications.

### 2.4 Statistical analysis

Data were analyzed using IBM SPSS Statistics (version 26.0; IBM Corp., Armonk, NY, USA). Continuous variables were presented as mean ± standard deviation, while categorical variables were summarized using frequencies and percentages. The normality of data distribution was assessed using the Shapiro–Wilk test. Group comparisons were performed using the Independent Samples *t*-test or the Mann–Whitney *U* test, as appropriate. Associations between categorical variables were evaluated using the Chi-square test. Exploratory factor analysis (EFA) was conducted on three items assessing parental awareness of myopia-related risk factors. The analysis revealed a single-factor structure explaining approximately 50% of the total variance. Factor loadings were acceptable, indicating a common underlying construct. However, the internal consistency of the scale was relatively low (Cronbach’s *α* ≈ 0.46), which may be attributed to the limited number of items and the exploratory nature of the questionnaire. Given that the questionnaire was designed for exploratory purposes rather than scale development, the results were considered sufficient to provide preliminary insights.

## 3 Results

Analyses were conducted on the questionnaire responses of 2,031 parents of children aged 3–17 years. Among the parents, 83.8% were female and 16.2% were male. The majority of parents were aged 36–45 years (54.4%), followed by 26.9% aged 26–35 years. Regarding educational background, 35.9% of parents were university graduates, and 48.8% had two children. The children included in the study were 51.7% male and 48.3% female. Age distribution was as follows: 46.5% were 7–10 years old, and 36.6% were 11–14 years old. According to parental reports, 74.7% of children had no ocular problems, while 11.8% were diagnosed with myopia. No history of myopia was reported in both parents in 53.5% of families, whereas 30.0% of families reported myopia in one parent.

Detailed demographic data are presented in Table 1.

**Table 1:** Demographic characteristics of the parents and children.

Variables	Groups	*n*	%
Parent gender	Female	1701	83.8
	Male	322	15.9
	20–25 years	20	1.0
	26–35 years	546	26.9
Parent age	36–45 years	1,104	54.4
	46–55 years	338	16.6
	56+ years	23	1.1
	Primary school	299	14.7
	Middle school	278	13.7
Parent education level	High school	591	29.1
	University	730	35.9
	Postgraduate	133	6.5
Number of children in the family	1 child	357	17.6
	2 children	991	48.8
	3 children	526	25.9
	**≥**4 children	157	7.7
Child age and education level	3–6 years [pre-school]	154	7.6
	7–10 years [primary school]	944	46.5
	11–14 years [middle school]	737	36.3
	15–17 years [high school]	186	9.2
	Other/not stated	10	0.5
Child gender	Female	972	48.3
	Male	1,042	51.7
Child vision status	Healthy	1,518	74.7
	Myopia	239	11.8
	Astigmatism	87	4.3
	Unknown	108	5.3
	Hypermetropia	23	1.1
	Other vision problems	56	2.8
Parental history of myopia	No myopia in both parents	1,087	53.5
	Myopia in 1 parent	610	30.0
	Myopia in 2 parents	127	6.3
	Unknown	188	9.3
	Other/Not stated	20	1.0

Daily screen time showed a distribution predominantly concentrated in moderate-to-high exposure categories, with 10.0% of children reporting <1 h/day, 34.5% reporting 1–2 h/day, and 34.6% reporting 3–4 h/day. When stratified by myopia status, higher screen exposure was more frequently observed among myopic children, with 69.1% falling within the 3–4, 5–6, and >6 h/day categories. A statistically significant association was identified between daily screen time and myopia (*p* < 0.001), indicating a positive relationship between increasing screen exposure and myopia prevalence. Accordingly, high screen use (≥3 h/day) was associated with a 1.94-fold increased risk of myopia (OR: 1.94).

When daily screen time was analyzed according to parental education level, children of parents with postgraduate education had the highest proportion of screen exposure <1 h per day (25.6%), whereas this rate was lowest among children of high school–educated parents (6.4%). The highest rate of daily screen time >6 h was observed in the high school parental group (13.5%). Across all education levels, the majority of children had daily screen exposure of 3–4 h, 5–6 h and more 6 h per day.

Screen time was also evaluated based on parental myopia status. In families where neither parent had myopia, 34.4% of children had 1–2 h of daily screen exposure and 34.0% had 3–4 h. In families with one myopic parent, 31.7% of children had 1–2 h of screen time, and 35.6% had 3–4 h. In the group with unknown parental myopia status, the corresponding rates were 31.9% for 1–2 h and 35.6% for 3–4 h. Age-stratified analysis indicated that children aged 3–6 years had the lowest proportion of high-risk screen exposure (3–4 h, 5–6 h and more 6 h per day) at 26.0%, whereas children aged 15–17 years had the highest risk at 84.4%. A significant positive correlation was observed between age and daily screen time (*p* < 0.001).

**Figure 1:**
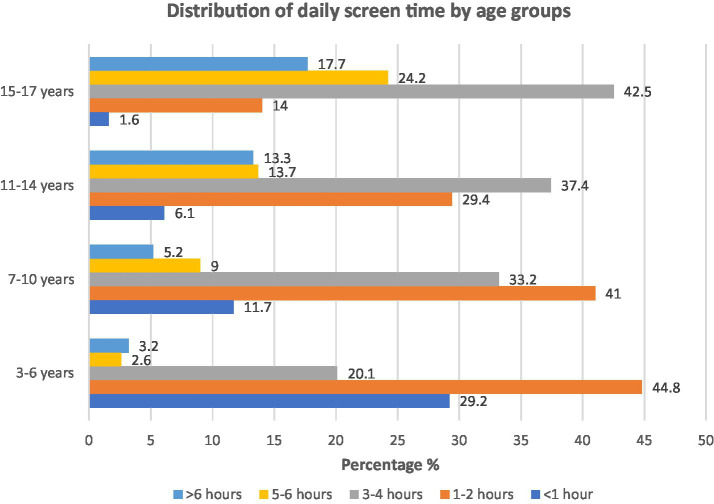
Distribution of daily screen time by age group (%).

Specifically, the proportion of children with screen time <1 h was lowest in the 15–17 years age group (1.6%) and highest in the 3–6 years age group (29.2%). Conversely, screen exposure 3–4 h, 5–6 h and more 6 h per day was most prevalent in the 15–17 years age group (84.4%). No significant differences were observed between males and females regarding daily screen time, with the highest exposure category (3–4 h, 5–6 h and more 6 h per day) comprising 52.4% of males and 58.5% of females. Detailed data are presented in Figure 1.

**Figure 2:**
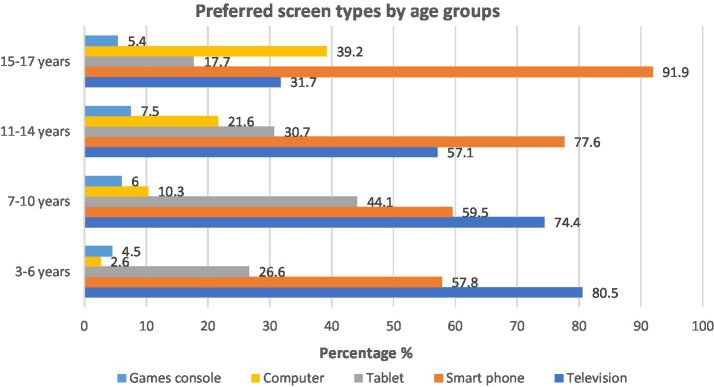
Distribution of preferred screen types by age group (%).

The types of screens used by children were reported as follows: smartphones (64.7%), televisions (59.2%), and tablets (30.6%). The most common combination was television plus smartphone (25.7%), whereas the least frequently used device was a gaming console. Regarding the number of devices used, 32.7% of children used a single screen, and 39.1% used two screens. When screen type usage was analyzed by age group, television predominated in children aged 3–6 years (80.5%), tablets in the 7–10 years group (44.1%), and in the 15–17 years group, computer use was 39.2% and smartphone use was 91.9%. With increasing age, television use decreased, while smartphone and computer use increased. Gaming console usage remained relatively stable across age groups, and tablet use increased up to the 7–10 years group, followed by a subsequent decrease. Detailed data are presented in Figure 2.

The most common combination of screen activities was playing games and watching videos, reported by 26.2% of children. Only watching videos was reported by 17.7%, only playing games by 10.6%, and screen use exclusively for lessons or homework by 2.2%. The combination of all three activities—lessons, games, and videos—was reported by 11.7% of children. When single activities were analyzed across all children, 74.0% engaged in watching videos, 64.3% in playing games, 31.9% in lessons or homework, and 15.5% in social media use. Detailed data are presented in Table 2.

**Table 2:** Distribution of children’s screen activity preferences (%).

Activity combinations	No.	%	Single activity	No.	%
Playing games + watching videos	532	26.2%	Watching videos	1,502	74.0%
Watching videos only	360	17.7%	Playing games	1,305	64.3%
Lessons/homework + games + watching videos	238	11.7%	Lessons/homework	647	31.9%
Playing games only	215	10.6%	Social media use	314	15.5%
Lessons/homework + watching videos	118	5.8%	Listening to music	1	0.0%
Lessons/homework + playing games	113	5.6%	Reading books/e-books	0	0.0%
Games+ video + social media	96	4.7%			
Lessons/homework + games + video + social media	65	3.2%			
Watching videos + social media	45	2.2%			
Lessons/homework only	44	2.2%			
Other combinations	242	11.9%			

Parental restrictions on screen use were evaluated. Moderate restrictions with basic rules were applied by 62.7% of parents, whereas stricter rules were implemented by 13.6%. A low level of restriction with minimal intervention was reported by 20.0% of parents, and 3.3% imposed no restrictions. Overall, the general tendency among parents was to adopt a balanced approach to screen control.

When restrictions were analyzed according to parental age, the highest overall restriction rate (98.5%) was observed in the 26–35 years age group, with a majority following basic rules (68.3%). Among parents who made no interventions, 25.0% were aged 16–25 years, and 13.0% were over 56 years.

Evaluation according to parental education level revealed that most parents applied moderate restrictions with basic rules. Only 10% of parents with postgraduate education imposed no restrictions. Age-related tendencies showed that the most flexible group was the 16–25 years age group, the most restrictive was the 26–35 years group, a balanced approach was observed in the 36–45 years group, and the 46–55 years and >56 years groups were generally more flexible. Regarding education level, parents with primary school and university education tended to impose stricter restrictions, those with middle school and high school education applied moderate restrictions, and postgraduate-educated parents were the most flexible.

Methods for managing screen use were also assessed. The most common method was limiting screen time (57.0%), followed by restricting content only (44.1%). Fourteen percent (14.2%) of parents did not implement any management strategies. The least commonly used method was engaging in activities together with the child (9.2%). Detailed data are presented in Table 3.

**Table 3:** Distribution of parental screen use management strategies (%).

Management combinations	No.	%	Single management preference	No.	%
Limited daily time only	376	18.5%	Daily time limit	1,158	57.0%
No method applied	289	14.2%	Content/application restriction	896	44.1%
Content restriction only	282	13.9%	Defined times for screen use	665	32.7%
Time limit + content restriction	241	11.9%	No method applied	289	14.2%
At defined times only	221	10.9%	Doing screen activities together	187	9.2%
Time limit + defined times	144	7.1%	Forbidding screen use at certain times	156	7.7%
Defined times + content restriction	130	6.4%			
Duration + time + content restriction	100	4.9%			
Doing activities together only	30	1.5%			
Other combinations	418	20.6%			

Parental concerns regarding their children’s screen use were evaluated. Psychological concerns, such as addiction, attention disorders, and behavioral problems, were reported most frequently (39.1%), followed by health-related concerns including eye health and postural issues (23.5%), concerns about inappropriate or harmful content (17.1%), educational concerns such as academic performance and learning difficulties (9.8%), and social concerns (5.8%). A small proportion of parents (3.7%) reported having no concerns.

Parental knowledge, attitudes, and practices regarding myopia were evaluated in a multidimensional manner. Overall, 44.5% of parents demonstrated a basic level of knowledge, 42.8% reported a good level, and 11.8% had no knowledge of myopia. When specific components of knowledge were examined, awareness of risk factors appeared higher than that of protective factors; 61.4% of parents believed that near work has a definite effect on myopia progression, whereas only 34.1% recognized the definite protective role of outdoor activities, and 29.1% reported no knowledge in this area. Despite this variability in knowledge, concern regarding screen time was high, with 60.5% of parents being very concerned and 36.4% somewhat concerned. Preventive practices were most commonly centered on behavioral modifications, including restricting screen time (80.0%) and encouraging regular eye examinations (61.2%), followed by ensuring proper lighting (47.8%) and promoting outdoor activity (42.8%). Notably, 34.2% of parents reported belief in the effectiveness of eye exercises. Overall, lifestyle-based strategies were more frequently perceived as effective (61.4%) compared to medical approaches (47.7%). In terms of the breadth of preventive practices, 24.7% of parents reported using three strategies, while 38.3% were aware of four or more, indicating variability in the comprehensiveness of approaches.

Outdoor activity patterns further supported these findings. The most commonly reported duration across all age groups was 1–2 h per day (31.5%). Adequate outdoor exposure (≥2 h/day) was more frequent among children aged 7–10 years (47.5%), whereas insufficient exposure (≤1 h/day) was most prevalent in the 3–6 years age group (31.2%). Regarding the balance between outdoor activities and screen use, 45.6% of parents reported partially achieving this balance, 38.9% reported fully achieving it, and 9.5% reported being unable to do so, with 84.5% overall indicating an attempt to maintain balance. The most commonly reported strategy was prioritizing schoolwork and allowing screen time after homework completion (69.6%), followed by a flexible, child-directed approach (12.7%) and prioritizing outdoor activities before screen use (11.1%).

Eye examination practices indicated that 42.9% of parents took their child for an eye examination only when symptoms occurred, 30.7% attended regular eye examinations, and 8.8% followed school screening recommendations. Differences were observed according to parental education level; regular eye examination rates were higher among parents with university (39.1%) and postgraduate education (37.3%), whereas parents with primary (24.4%) and middle school education (19.4%) more frequently reported that their children had never undergone an eye examination.

The frequency of regular eye examinations showed variation according to family history of myopia, with higher rates observed among children with a stronger familial predisposition. Regular eye examination rates were 38.6% when both parents were myopic, 36.0% when one parent was myopic, and 29.3% when neither parent had myopia, suggesting a potential positive association between familial myopia burden and preventive eye care utilization.

Among parents who had never taken their child for an eye examination, a substantial proportion reported uncertainty regarding their own myopia status (23.5%), while 16.9% reported no myopia and 12.3% reported that one parent was myopic, indicating that lack of awareness of parental refractive status may be associated with lower uptake of eye examinations.

Among children who attended regular eye examinations, refractive errors were frequently identified, with myopia detected in 39.3% and astigmatism in 36.8%, while 28.6% were reported to have normal vision, indicating that routine examinations were largely associated with the detection of clinically relevant visual conditions. In contrast, visual status remained uncertain in a notable proportion of children who had not undergone eye examinations (24.1%), while 17.0% were reported as having normal vision, reflecting limited objective assessment in this group.

The indications for the most recent eye examination were predominantly preventive, as routine check-ups constituted the main reason for attendance (42.3%). Symptom-driven visits were less common, including eye pain or itching (12.5%), school-based detection of vision problems (7.4%), and squinting (4.4%). However, a substantial proportion of children (27.5%) had never undergone an eye examination, indicating a persistent gap in preventive eye care utilization.

Parental knowledge of myopia-related topics showed an uneven distribution across different domains, indicating variability in both depth and breadth of awareness. Knowledge was highest for risk factors such as inadequate lighting and prolonged near work (46.4%), followed by awareness of corrective glasses (42.4%), whereas recognition of the protective role of outdoor activity was lower (30.1%) and knowledge of pharmacological treatment options was particularly limited (14.9%), suggesting a shift from general to more specialized knowledge domains. Notably, 26.9% of parents reported no knowledge across any of the assessed areas, indicating a substantial subgroup with complete lack of awareness. When evaluated in terms of overall knowledge structure, most parents demonstrated only partial understanding, with 42.3% exhibiting moderate knowledge (two to three domains) and 24.5% demonstrating comprehensive knowledge across all four domains. Although 73.1% of parents were aware of at least one aspect of myopia management, the marked decline in awareness across progressively specific domains suggests that parental knowledge is largely fragmented rather than comprehensive.

## 4 Discussion

This study represents one of the first comprehensive investigations among Turkish parents of children aged 3–17 years examining knowledge, attitudes, and behaviors related to childhood myopia, screen use, and protective factors. A total of 2,031 parents participated. Although 87.3% of parents reported at least basic knowledge of myopia, only 24.5% demonstrated comprehensive knowledge, while 26.9% reported no knowledge, indicating a marked discrepancy between general awareness and in-depth understanding. This pattern suggests that parental knowledge is largely superficial and not uniformly distributed across different domains of myopia-related information.

When compared with previous findings, the overall awareness level in this study was higher than that reported by Huang et al. (21) in China (68.2%), which may reflect differences in population characteristics or health education exposure. However, despite this relatively high baseline awareness, the low proportion of comprehensive knowledge highlights persistent deficiencies, particularly in more specific areas such as protective factors and management strategies. Overall, these findings indicate that while myopia is generally recognized by parents, detailed and actionable knowledge remains insufficient.

Risk factor awareness was relatively high among parents, with 87.1% recognizing the role of close work and screen use in myopia development, possibly reflecting effective dissemination of risk-focused public health messages. In contrast, awareness of protective factors was more limited, as only 62.4% identified the protective role of outdoor activities and 29.1% reported no knowledge in this area, indicating a clear imbalance between risk-oriented and prevention-oriented understanding. This pattern is consistent with previous literature, as Qian and Lu (22) reported that 42.3% of parents were unaware of the protective effect of outdoor activity, further supporting that knowledge of preventive strategies remains insufficient across populations. Overall, these findings suggest that while awareness of risk factors is well established, understanding of protective behaviors is comparatively underdeveloped and represents a key gap in parental myopia-related health literacy.

Knowledge of treatment options was also limited. While 42.4% of parents were aware of corrective glasses for myopia, only 14.9% were familiar with pharmacological interventions such as low-dose atropine. Similarly, a study conducted in Spain reported that 31.7% of parents were aware of myopia control methods (23). These findings indicate that, despite a relatively high level of general awareness, knowledge regarding contemporary treatment strategies remains insufficient. This gap highlights the need for a more structured and systematic integration of parental education into primary healthcare and ophthalmology services.

The present study confirmed that a substantial proportion of children exceed recommended daily screen time limits. Consistent with these findings, Huang et al. demonstrated that early-life screen exposure increases the risk of developing myopia, whereas time spent outdoors has a protective effect (24). Additionally, engaging in near work at distances of less than 20 cm for up to 2 h per day has been identified as a significant risk factor (25, 26). Supporting this, Fu et al. (27) reported that shorter viewing distances may accelerate myopia progression through mechanisms such as hypermetropic retinal defocus (28). Taken together, these findings suggest that both prolonged screen exposure and close viewing distances play a critical role in myopia development and progression. From a public health perspective, these results highlight the importance of promoting healthy screen habits, encouraging outdoor activities, and increasing parental awareness as key strategies for myopia prevention in children.

In the current study, intensive screen use (≥3 h/day) was associated with a 1.94-fold increased risk of myopia (*p* < 0.001). Consistent with our findings, Ha et al. reported a 21% increase in myopia risk per additional hour of daily screen time in a meta-analysis of 45 studies (20), while Alvarez-Peregrina et al. demonstrated significantly greater screen exposure among myopic children (29). However, it is important to interpret these findings with caution, as myopia status in our study was determined solely based on parental reports rather than objective clinical measurements. This reliance on subjective assessment may have introduced misclassification bias, potentially leading to either overestimation or underestimation of the true association between screen time and myopia. Therefore, although our results support the existing body of evidence, they should be validated by future studies incorporating cycloplegic refraction and standardized ophthalmologic examinations.

A key finding of this study was the marked discrepancy between parental knowledge and actual behavior. Although 96.3% of parents reported restricting screen use, more than half of the children (55.5%) still exceeded 3 h per day. Given that the World Health Organization recommends a maximum of 2 h/day for children older than 5 years, this prevalence is notably high (30, 31). This inconsistency, also reported by Alvarez-Peregrina et al. (29), reflects the so-called “intention–behavior gap”, which describes the disconnect between individuals’ stated intentions and their actual behaviors. In the context of our findings, despite high levels of parental awareness and expressed intent to limit screen exposure, these intentions do not consistently translate into effective preventive actions. Several interrelated factors may explain why awareness alone fails to result in behavioral change. First, parents may lack practical strategies and structured monitoring approaches, leading to difficulties in consistently regulating children’s screen use. Supporting this, 68.6% of children in our study used multiple screen devices, making it challenging to accurately estimate cumulative exposure, as different devices are often considered separately rather than as part of total daily screen time. Second, environmental and contextual constraints play a substantial role; the integration of digital devices into education, entertainment, and social interaction has reduced the feasibility of strict restrictions. Third, behavioral and psychological factors, including children’s resistance to screen limitations and parental fatigue or competing responsibilities, may further hinder the implementation of intended rules. Additionally, screen use for educational purposes is frequently perceived as harmless, which may lead to underestimation of total exposure and more permissive attitudes. The normalization of elevated screen use following the COVID-19 pandemic may have further reinforced these patterns; a 2025 meta-analysis by Ha et al. (20) reported a 33% increase in children’s screen time, with persistently high levels thereafter. Collectively, these findings suggest that awareness alone is insufficient to drive meaningful behavioral change. Therefore, interventions should extend beyond knowledge dissemination and focus on enhancing parental self-efficacy, promoting structured monitoring strategies, and modifying the home environment to support sustainable reductions in screen time.

A similar discrepancy between awareness and behavior was observed regarding outdoor activities. Although 62.4% of parents were aware of the protective effect of outdoor exposure against myopia, only 44.0% reported that their child spent more than 2 h per day outdoors. This pattern mirrors the previously described gap between parental knowledge and actual behavioral implementation observed for screen use, suggesting a consistent failure of awareness to translate into preventive action. The importance of outdoor exposure in myopia prevention has been well documented in the literature. French et al. (11) reported that at least 2 h of daily outdoor activity may reduce the risk of developing myopia by up to 50%, while Alvarez-Peregrina et al. (29) demonstrated significantly lower myopia rates among children with regular outdoor exposure compared with those without. Similarly, Huang et al. (21) found that 56.4% of children spent less than 1 h per day outdoors, with even lower exposure among myopic individuals. Supporting these findings, French et al. (13) also reported a clear difference in weekly outdoor exposure between myopic (16.3 h/week) and non-myopic children (21.0 h/week). Guo et al. (14) further confirmed a dose-dependent inverse relationship between outdoor time and myopia prevalence, and Sherwin et al. (15) quantified this effect, showing that each additional hour of outdoor activity per week was associated with a 2% reduction in the probability of myopia. Collectively, these findings reinforce outdoor exposure as a consistent and modifiable protective factor; however, its limited translation into daily behavior in real-world settings, alongside similar patterns observed for screen use, highlights a broader and persistent intention–behavior gap in parental implementation of myopia-related preventive strategies. The findings of the present study further suggest that awareness alone is insufficient to ensure meaningful behavioral change. Beyond individual knowledge, a range of structural and contextual barriers appear to limit the translation of awareness into practice. These include restricted access to safe outdoor environments, increasing academic demands, and time constraints related to parental work schedules, all of which may collectively hinder the implementation of recommended preventive behaviors. This underscores the need for a broader, systems-level approach in myopia prevention strategies that addresses not only parental awareness but also the environmental and social conditions shaping children’s daily behaviors.

Parental education level was strongly associated with knowledge of myopia, adoption of protective behaviors, and utilization of eye care services. Regular eye examination rates were substantially higher among parents with postgraduate education (39.1%) compared with those with primary school education (15.7%), indicating a clear gradient in preventive health engagement. Similarly, higher educational attainment was associated with more consistent encouragement of outdoor activities and stricter regulation of screen time exposure. In contrast, a notable proportion of children of low-education parents (24%) had never undergone an eye examination, compared with only 3% among those with highly educated parents, further underscoring disparities in access and utilization of preventive care. These findings suggest that parental education, as a key component of health literacy, plays a central role in shaping both awareness and implementation of myopia-related preventive strategies. Beyond individual knowledge, this association likely reflects broader socioeconomic inequalities that influence access to healthcare services, interpretation of health information, and prioritization of preventive behaviors. Accordingly, these results highlight the need for targeted public health interventions and educational strategies specifically designed to reach socioeconomically disadvantaged groups in order to reduce disparities in childhood eye health.

Parents with a family history of myopia appeared to be somewhat more proactive in adopting preventive behaviors, with regular eye examination rates of 38.6% when both parents were myopic, compared with 29.3% among families with no reported history. These findings may suggest that personal exposure to myopia within the family increases parental awareness and may be associated with greater engagement in eye care practices. However, this association should be interpreted with caution, as the study design and reliance on self-reported data limit the ability to draw strong causal inferences regarding the effect of genetic predisposition on preventive behaviors. In particular, family history was not clinically verified, and behavioral outcomes were based on parental reporting, which may introduce reporting bias. Therefore, while our findings are broadly consistent with previous reports such as Qian and Lu (22), they primarily indicate a potential relationship rather than a definitive effect of genetic predisposition on parental behavior.

A concerning finding of the present study was that 27.5% of parents had never taken their child for an eye examination, a proportion substantially higher than that reported in many developed countries (approximately 5–10%). This suggests potential gaps in awareness, accessibility, or prioritization of preventive eye care within the study population. Only 30.7% of children underwent regular eye examinations, a rate comparable to findings from China (28.2%) (21), indicating that low adherence to routine screening may be a broader international issue rather than a region-specific phenomenon. Notably, 42.9% of examinations were symptom-driven, which may contribute to delayed detection of refractive errors, including myopia, and consequently postpone timely intervention. This reactive pattern of healthcare utilization reflects limited engagement in preventive eye health practices. In addition, school-based screening accounted for only 7.4% of eye examinations, suggesting a limited contribution of existing school health programs to early detection. Collectively, these findings underscore the need not only to increase awareness but also to strengthen structured and proactive screening systems, particularly through the expansion and improvement of school-based eye health programs, to facilitate earlier identification and management of myopia.

Age-specific differences in screen use patterns were clearly observed across the study population. Television use was predominant among younger children aged 3–6 years (80.5%), whereas smartphone use increased markedly with age, reaching 91.9% in adolescents aged 15–17 years. Similarly, computer use showed an age-dependent rise, reaching 39.2% among high school students. Importantly, high-risk screen exposure (≥3 h/day) peaked in the oldest age group (84.4%), which corresponds to a critical developmental period for myopia progression. These findings suggest that as children age, screen use shifts from passive to more interactive and multifunctional devices, which may increase both exposure duration and intensity. The observed pattern is likely influenced by increasing academic requirements, greater engagement in social media, and entertainment-driven digital activities. In adolescence, reduced parental supervision further limits external control over screen behavior, shifting responsibility increasingly to the individual. This transition highlights the need for interventions that extend beyond parental regulation and instead focus on developing self-regulation and digital self-management skills, particularly during adolescence when screen exposure is highest and potentially most impactful on myopia development.

The timing of this study in 2025 is particularly relevant for assessing the long-term behavioral impact of the COVID-19 pandemic. The persistently high rates of screen use observed in the present cohort suggest that the pandemic may have contributed not only to a temporary increase in digital exposure but also to more sustained behavioral shifts. The widespread adoption of online education, remote social interaction, and digital entertainment appears to have normalized prolonged screen use in children’s daily routines. This normalization may have altered parental perceptions of what constitutes “acceptable” screen exposure, potentially shifting pre-pandemic behavioral benchmarks. As a result, behaviors that were previously considered excessive may now be perceived as routine or unavoidable. These findings indicate that addressing screen-related risk factors may require strategies beyond individual-level education, and instead necessitate broader societal and policy-level interventions aimed at redefining healthy digital behavior standards in the post-pandemic era.

Several limitations of this study should be considered when interpreting the findings. First, the cross-sectional design limits the ability to establish causal relationships; therefore, the observed associations between screen time, outdoor activity, and myopia can only be interpreted as correlations rather than evidence of causality.

Second, myopia status was not clinically confirmed through objective ophthalmologic examination, including cycloplegic refraction. Instead, myopia was based on parental report, which may introduce misclassification bias and affect the accuracy of prevalence estimates as well as the observed associations with behavioral and environmental factors.

Third, the study relied on parent-reported data for key behavioral variables, including screen time and outdoor activity, which may introduce reporting and recall bias. Parents may have underestimated or inaccurately reported their children’s actual exposure levels, potentially leading to differential misclassification and attenuation of true associations. In addition, the analyses were primarily based on univariate statistical tests (chi-square and *t*-tests), which limits adjustment for potential confounding variables. Considering the multifactorial etiology of myopia, factors such as age, parental myopia, and socioeconomic status may have influenced the observed associations. Therefore, the findings should be interpreted as unadjusted associations rather than independent effects. Future studies using multivariable regression models are needed to better account for confounding and to validate the robustness of these relationships.

Finally, the use of an online survey introduces potential selection bias, as families without reliable internet access, adequate digital literacy, or engagement with online platforms may have been excluded. This may result in underrepresentation of lower socioeconomic groups and limit the generalizability of the findings to the broader population.

## 5 Conclusion

Considering that parents significantly influence the health and treatment processes of their children, understanding their knowledge, attitudes, and practices regarding myopia is essential. This understanding provides insight into parental perspectives and enables clinicians to develop more effective public education initiatives and myopia control strategies. The findings of this study reinforce the dominant role of genetic predisposition (family history) in childhood myopia. Among environmental factors, managing screen exposure and increasing time spent outdoors emerge as the primary modifiable risk factors. The relatively low sensitivity of the model indicates that additional, as-yet-unidentified factors contribute to the development of myopia, highlighting the need for further, comprehensive research in this field.

## Data Availability

The original contributions presented in the study are included in the article/supplementary material, further inquiries can be directed to the corresponding author.
